# Beryllium Stress-Induced Modifications in Antioxidant Machinery and Plant Ultrastructure in the Seedlings of Black and Yellow Seeded Oilseed Rape

**DOI:** 10.1155/2018/1615968

**Published:** 2018-03-21

**Authors:** Skhawat Ali, Rong Jin, Rafaqat A. Gill, Theodore M. Mwamba, Na Zhang, Zaid ul Hassan, Faisal Islam, Shafaqat Ali, Weijun Zhou

**Affiliations:** ^1^Institute of Crop Science and Zhejiang Key Laboratory of Crop Germplasm, Zhejiang University, Hangzhou 310058, China; ^2^Agricultural Experiment Station, Zhejiang University, Hangzhou 310058, China; ^3^Oil Crops Research Institute, Chinese Academy of Agricultural Sciences, Wuhan 430062, China; ^4^Department of Environmental Sciences and Engineering, Government College University, Faisalabad 38000, Pakistan

## Abstract

Beryllium (Be) could be a threatening heavy metal pollutant in the agroecosystem that may severely affect the performance of crops. The present study was conducted to evaluate the toxic effects of Be (0, 100, 200, and 400 *μ*M) on physiological, ultrastructure, and biochemical attributes in hydroponically grown six-day-old seedlings of two cultivars of* Brassica napus* L., one tolerant (ZS 758, black seeded) and one sensitive (Zheda 622, yellow seeded). Higher Be concentrations reduced the plant growth, biomass production, chlorophyll contents, and the total soluble protein contents. A significant accumulation of ROS (H_2_O_2_, OH^−^) and MDA contents was observed in a dose-dependent manner. Antioxidant enzymatic activities including SOD, POD, GR, APX, and GSH (except CAT) were enhanced with the increase in Be concentrations in both cultivars. Relative transcript gene expression of above-mentioned antioxidant enzymes further confirmed the alterations induced by Be as depicted from higher involvement in the least susceptible cultivar ZS 758 as compared to Zheda 622. The electron microscopic study showed that higher level of Be (400 *μ*M) greatly damaged the leaf mesophyll and root tip cells. More damage was observed in cultivar Zheda 622 as compared to ZS 758. The damage in leaf mesophyll cells was highlighted as the disruption in cell wall, immature nucleus, damaged mitochondria, and chloroplast structures. In root tip cells, disruption in Golgi bodies and damage in cell wall were clearly noticed. As a whole, the present study confirmed that more inhibitory effects were recorded in yellow seeded Zheda 622 as compared to black seeded ZS 758 cultivar, which is regarded as more sensitive cultivar.

## 1. Introduction

Oilseed rape (*Brassica napus *L., AACC genome) has developed through allopolyploids between two diploid parents,* Brassica rapa* (AA genome) and* B. oleracea *(CC genome) [1].* B. napus* belongs to the family Brassicaceae which has been extensively used for the phytoextraction [2].* Brassica *species have become the second largest oil producing crop since the last decade [3]. These species have greater potential to grow well under heavy metal (HM) contaminated soils because of their higher metal tolerance ability [4]. Due to these unique properties,* B. napus *has gained the attention of researchers in recent years [5].

HM toxic effects in the surrounding environment impose severe threats to agricultural crop production and quality by reducing the plant growth and yield [6, 7]. The excessive HM concentrations reduce the seed germination [8] and photosynthesis and cause nutrient balance, root damage, and ultimately plant death [6, 9]. The oxidative stress induced by HMs increases the production of reactive oxygen species (ROS) [8]. To cope with these ROS, plants have developed antioxidant scavenging system in the form of total superoxide dismutase (SOD), peroxidase (POD), catalase (CAT), ascorbate peroxidase (APX), glutathione reductase (GR), and the contents of reduced glutathione (GSH), oxidized glutathione (GSSG), and total glutathione (GSH + GSSG) [10]. Furthermore, HM stresses significantly alter the cellular protein functions and thylakoid membrane structures, which are directly associated with plant photosystem [11–13]. In addition, HMs impair lipid composition of cell membrane [14, 15] and higher accumulation in agricultural soil system results in health hazards due to their direct entry into food chain [16].

Beryllium (Be) is the element of group IIA in periodic table with an average concentration of 2.8–5 mg/kg on the earth's crust [17]. It has gained worldwide economic attention due to its extensive use in nuclear weapons, reactors, X-ray machines, electronic industries, and aircraft structures [18]. However, its entry into the environment has become alarming for the sustainability of the ecosystem [19]. Major entry sources of Be into our ecosystem are fossil fuel burning, industrial discharge, and atmospheric emission [16]. Be is readily taken up by plants and accumulates into their edible parts [20].

It has been found that higher levels of Be appreciably declined the growth of soybean young seedlings [21] and yield reduction by 50% in cabbage [22]. In addition, it reduces the seed germination, root length, and dry weight in various plants [23]. Recently, Agrawal et al. [24] found that Be significantly enhanced the lipid peroxidation rate and reduced glutathione level and antioxidant enzyme activities. Inorganic salts of Be, such as BeCl_2_, are generally more toxic to plants, mainly because of their high solubility. Moreover, Encina and Becerra [25] proposed that a particular level of membrane-associated Ca is essential for the fusion of Golgi vesicles in the cell plate. The presence of Be might displace the Ca from its binding sites, which can hinder the cell formation.

*Brassica* species have potential to tolerate against HM stresses. Therefore, it is imperative to evaluate the responses and mechanism of these species against Be stress. It has been discussed previously that Be declined the plant growth and yield by inhibiting morphology, physiology, and biochemical processes [19, 26]. Since limited data is available regarding morphological and physiobiochemical responses of plants to Be-toxicity in* Brassica* species, the present study was carried out to understand the growth, photosynthesis, oxidative stress, and antioxidant and ultrastructural modifications induced by Be-toxicity in response to two* B. napus *cultivars, that is, ZS 758 (black seeded, tolerant) and Zheda 622 (yellow seeded, sensitive).

## 2. Materials and Methods

Two potential cultivars (ZS 758, black seeded; Zheda 622, yellow seeded) of oilseed rape* (B. napus)* used in the present study were selected on the basis of our previous study [6] in which these cultivars showed significant differences in their metal tolerance ability. Good quality and mature seeds were obtained from the College of Agriculture and Biotechnology, Zhejiang University. At first, seeds were treated with 70% (v/v) ethanol for 3 min, transferred into 0.1% (m/v) HgCl_2_, and then rinsed with deionized water thoroughly. A total of 40 seeds were positioned in every Petri dish on wet filter paper for overnight. After germination, 25 seedlings were randomly chosen for every treatment and then transferred to plastic Petri dishes (12 cm^2^) with two pieces of filter papers lying on sponge. To which, 6 mL of beryllium (Be) solutions (0, 100, 200, and 400 *μ*M) were added. After 24 h, the excessive solution was discarded and seedlings were treated with half-strength Hoagland's solution. Beryllium sulphate (BeSO_4_) salt was used to maintain different Be concentrations. Four replications per treatment were taken in full-strength Hoagland's solution. Seedlings were allowed to grow in controlled conditions with day/night temperatures of 25/20°C, a 16-h photoperiod, an irradiance of 300 *μ*mol m^−2^ s^−1^, and a relative humidity of 60–70%. After 8 days of treatment, seedlings were harvested and separated into shoots and roots for the determinations of morphological, ultrastructural, and biochemical characteristics.

Plant growth characteristics regarding the shoot and root lengths were measured. Fresh and dry biomasses of plant parts, that is, leaf, stem, and root, were measured according to Zhang et al. [27]. The method employed by Porra et al. [28] was followed to analyze the chlorophyll (Chl a, b) and carotenoids (Car) contents.

Lipid peroxidation in* B. napus* seedlings was analyzed regarding malondialdehyde (MDA) by following the procedure of Zhou and Leul [29]. Fresh samples (0.5 g) of leaves and roots were extracted in 8 mL of 0.25% thiobarbituric acid (TBA) in 10% trichloroacetic acid (TCA). Then the extract was heated at 95°C for 30 min and then cooled on ice. After this, the samples were centrifuged at 5,000 ×g for 10 min, and absorbance was checked at 532 nm. The level of MDA was expressed as mmolg^−1^ protein by using extinction coefficient (155 mM cm^−1^). Hydrogen peroxide (H_2_O_2_) contents were measured according to Gong et al. [30]. The samples (0.5 g) were treated with 0.1% (w/v) trichloroacetic acid (TCA) (5 mL) in ice bath. The homogenized samples were centrifuged for 15 min at 12,000 ×g (Eppendorf AG, model 2231, Hamburg, Germany). Then, supernatant (1.5 mL) was mixed with 0.5 mL of 10 mM potassium phosphate buffer (pH 7.0) and 1 M KI (1 mL). The absorbance was taken at 390 nm, and H_2_O_2_ contents were calculated by using a standard curve [31]. For the quantification of hydroxyl radicals (OH^−^), 0.5 g fresh samples were treated with 3 mL of 10 mM Na-phosphate buffer (pH 7.4) consisting of 15 mM 2-deoxy-D-ribose (SRL, Mumbai) at 37°C for 2 h [32]. After this, an aliquot of 0.7 mL of the above-homogenized samples were added to a reaction mixture containing 3 mL of 0.5% (w/v) thiobarbituric acid (TBA), 1% stock solution made in 5 mM NaOH, and 1 mL glacial acetic acid, then heated at 100°C in a water bath for 30 min, and cooled down to 41°C for 10 min. Absorbance was checked at 550 nm by using a spectrophotometer.

Law et al. [33] method was followed to estimate the reduced glutathione (GSH) and oxidized glutathione (GSSG) in plant samples. 0.5 g samples were treated with 5 mL of 10% (w/v) trichloroacetic acid (TCA) and then centrifuged at 15,000 ×g for 15 min. For the estimation of GSH contents, the supernatant of 0.150 mL was mixed with 100 *μ*L of 6 mM dithionitrobenzoate (DTNB), 50 *μ*L of glutathione reductase (10 units mL^−1^), and 0.7 mL of 0.3 mM NADPH. To measure GSSG, the supernatant of 0.120 mL was combined with 0.010 mL of 2-vinylpyridine followed by the addition of 0.020 mL of 50% (v/v) triethanolamine. Then, the solution was well-mixed with vortex for the 30 s and incubated at 25°C for 25 min. At the end, reduced glutathione contents were determined by subtracting GSSG from the total glutathione content.

Antioxidant enzyme activities were measured according to Zhang [34] with some modifications. Fresh samples (0.5 g) were ground in 8 mL of 50 mM potassium phosphate buffer (pH 7.8) under cold conditions, then homogenized, and centrifuged at 10, 000 g at 4°C for 20 min. After this, the supernatant was taken for the enzymatic assays [35]. Total superoxide dismutase (SOD) activity was assessed by following the inhibition of photochemical reduction of nitroblue tetrazolium (NBT) [27]. Reaction mixture was comprised of 50 mM potassium phosphate buffer (pH 7.8), 0.075 mL NBT, 0.002 mL riboflavin, 13 mM methionine, 0.1 mM EDTA, and 0.100 mL of enzyme extract in a 3 mL volume. One unit of SOD activity was measured as the amount of enzyme required to cause 50% inhibition of NBT reduction measured at 560 nm. Peroxidase (POD) activity was assayed by following Leul and Zhou [35] with some modifications. The reactant mixture contained 50 mM potassium phosphate buffer (pH 7.0), 0.4% H_2_O_2_, 1% guaiacol, and 0.1 mL enzyme extract. Variation due to guaiacol was measured at 470 nm. Catalase (CAT) activity was assayed with the use of H_2_O_2_ (extinction coefficient 39.4 mM cm^−1^) for 1 min at 240 nm in 3 mL reaction mixture containing 50 mM potassium phosphate buffer (pH 7.0), 10 mM H_2_O_2_, 2 mM EDTA-Na2, and 0.1 mL enzyme extract [36]. The ascorbate peroxide (APX) activity was estimated in a reaction solution comprised of 100 mM phosphate (pH 7), 0.06 mM H_2_O_2_, 0.3 mM ascorbic acid (AsA), 0.1 mM EDTA-Na_2_, and 0.1 mL enzyme extract [37]. The absorption was measured at 290 nm after the addition of H_2_O_2_. Glutathione reductase (GR) activity was determined according to Jiang and Zhang [38] with the oxidation of NADPH for 1 min at 340 nm (extinction coefficient 6.2 mM cm^−1^). The reaction mixture was comprised of 50 mM potassium phosphate buffer (pH 7.0), 0.15 mM NADPH, 2 mM EDTA-Na_2_, 0.5 mM GSSG, and 0.1 mL enzyme extract in a 1 mL volume. Total soluble protein (TSP) content was measured by following the method of Bradford [39]. Bovine serum albumin was used as a standard.

Total RNA was extracted from ~100 mg frozen leaf and root tissues using manual (Trizol) method. To remove the genomic DNA and cDNA synthesis, prime Script™ RT reagent kit (Takara, Co. Ltd., Japan) with gDNA (genomic DNA) eraser was used. cDNA samples from different treatments were assayed by quantitative real-time PCR (qRT-PCR) in the iCycleriQTM real-time detection system (Bio-Rad, Hercules, CA, USA) by using SYBRR Premix Ex Taq II (Takara, Co. Ltd., Japan). The software given with the PCR system was used to calculate the threshold cycle values [40].  Table S1 summarizes the specific primers used for each gene.

For ultrastructural analysis of leaf, fragments without veins (about 1 mm^2^) and root tips (about 2-3 mm) were fixed in 2.5% glutaraldehyde (v/v) in 0.1 M potassium phosphate buffer (PBS, pH 7.4) overnight and washed three times with the same PBS. Later, samples were postfixed in 1% OsO_4_ [osmium (VIII) oxide] for 1 h and then washed three times in 0.1 M PBS (pH 7.4) with 10-min intervals between each washing. After 15–20-min, the samples were dehydrated in a graded series of ethanol (50%, 60%, 70%, 80%, 90%, 95%, and 100%) and then used the absolute acetone for 20 min. The samples were infiltrated and embedded in Spurr's resin overnight. After heating at 70°C for 9 h, ultrathin sections (80 nm) of specimens were prepared and mounted on copper grids for the observation in transmission electron microscope (JEOL TEM-1230EX, Tokyo, Japan) at an accelerating voltage of 60.0 kV.

Analysis of variance (ANOVA) was carried out by using statistical analysis package SPSS, version 16.0 (SPSS, Chicago, IL, USA); differences were considered significant at *P* < 0.05. Data are the means ± standard deviation (SD) of three independent replicates. Significant means were compared by following Duncan's multiple range test.

## 3. Results

Plant growth characteristics regarding the shoot height, root elongation, and biomass production were severely affected by Be stress in a dose-dependent manner in both cultivars (Table 1). Be-induced deleterious effects were also evident even at the lowest dose (100 *μ*M), except for shoot height and stem fresh weight in ZS 758, as well as root fresh weight in both ZS 758 and Zheda 622. In recent studies, we reported a differing level of metal tolerance ability of cultivars ZS 758 and Zheda 622 under Cr [6], Cu, and Cd [41] stress. However, in this study, a relatively less difference was observed in the sensitivity and tolerance ability of these cultivars against Be stress. At the exposure of different Be concentrations (100, 200, and 400 *μ*M), no big genotypic difference was observed among all growth parameters except for dry leaf weight (Table 1). The detrimental effects of Be were evidenced on chlorophyll and carotenoids contents in both cultivars of* B. napus* leaves (Table 2). Less obvious deleterious effects were noticed in chlorophyll contents at the lowest level of Be exposure (100 *μ*M). The chlorophyll contents (Chl a, b) were decreased significantly with the increase in Be levels in both cultivars. A clear genotypic difference was observed at 200 and 400 *μ*M Be levels, contrary to the morphological observations. However, highest level of Be (400 *μ*M) was more detrimental to chlorophyll contents irrespective of the cultivars used. The levels of total soluble protein in the leaves of* B. napus* seedlings were gradually reduced with the increase in Be concentrations. Nonsignificant genotypic differences were noted at lower Be concentration (100 *μ*M), in comparison with 200 and 400 *μ*M (Table 2). Overall, higher protein levels were prominent in ZS 758 as compared to Zheda 622.

The accumulation of ROS and the subsequent oxidation of lipids in terms of MDA contents are the reliable indication of cellular damage occurred in plants. The exposure of* B. napus *seedlings to elevated levels of Be caused a marked induction of ROS (H_2_O_2_ and OH^−^) and MDA contents in the root and leaf tissues of both cultivars (Table 3). At 100 *μ*M Be level, sufficient contents of ROS were induced in both root and leaf tissues. A reduction in the accumulation of MDA contents was prominent at 200 and 400 *μ*M. The accumulation of OH^−^ contents was insignificant at 100 *μ*M Be in leaves. In roots, a significant difference was obvious at the highest Be dose (400 *μ*M). Similar trends in the accumulation of ROS and MDA contents were noted in both cultivars. It was apparent that Zheda 622 was more susceptible to Be stress as compared to ZS 758, which is evident from the induction of H_2_O_2_, OH^−^, and MDA contents.

Plants develop nonenzymatic antioxidant system against HM stress in the form of glutathione (GSH), which is the primary detoxifying system [42]. The alterations in the GSH and GSSG contents were observed in the leaves and roots of* B. napus* cultivars under various levels of Be (Table S2). A marked increase in GSH and GSSG contents was observed in both* B. napus* cultivars in a dose-dependent manner. At 200 and 400 *μ*M Be levels, higher GSH and GSSG contents were observed as compared with the control and 100 *μ*M. At 400 *μ*M Be level, maximum increase in both GSH and GSSG contents was observed in the leaves and roots of* B. napus* cultivars. Intermediate values of these contents were noted at 200 and 400 *μ*M Be levels.

**Figure 1:**
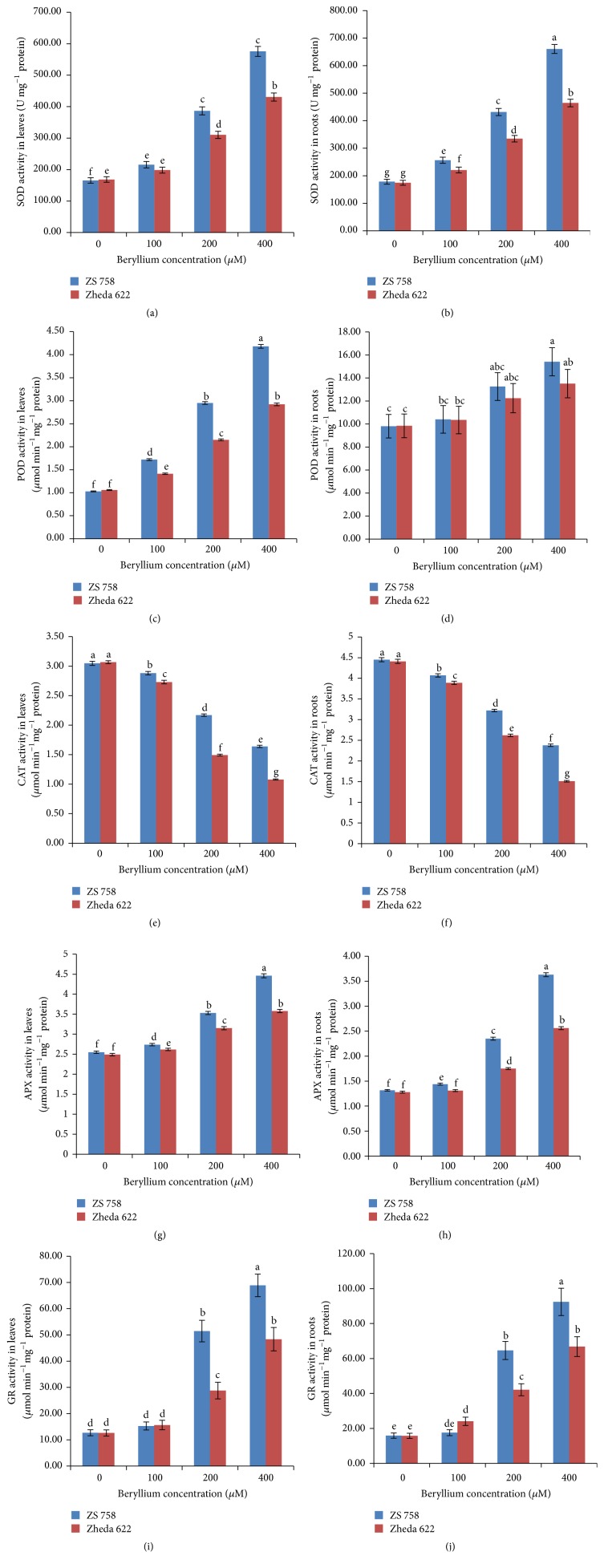
Effects of different concentrations of beryllium (Be) (0, 100, 200, and 400 *μ*M) on the activities of (a, b) superoxide dismutase (SOD), (c, d) guaiacol peroxidase (POD), (e, f) catalase (CAT), (g, h) ascorbate peroxidase (APX), and (i, j) glutathione reductase (GR), respectively, in the leaves and roots of 6-day-old seedlings of two* Brassica napus *cultivars (ZS 758, black seeded; Zheda 622, yellow seeded). Vertical bars represent standard deviation from three independent replicates. Means followed by the same letters are not significantly different by Duncan's multiple range test (*P* < 0.05).

**Figure 2:**
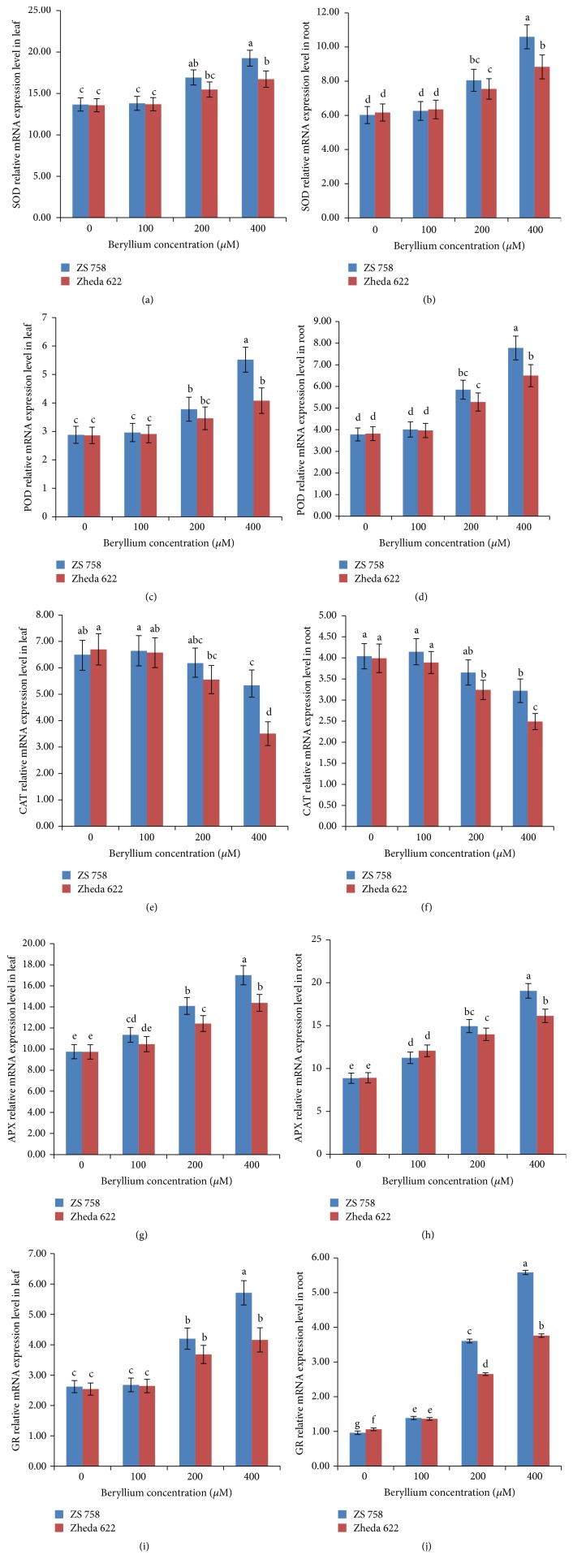
Effects of different concentrations of beryllium (Be) (0, 100, 200, and 400 *μ*M) on the transcript level of (a, b) superoxide dismutase (SOD), (c, d) guaiacol peroxidase (POD), (e, f) catalase (CAT), (g, h) ascorbate peroxidase (APX), and (i, j) glutathione reductase (GR) related gene expression, respectively, in the leaves and roots of 6-day-old seedlings of two* Brassica napus *cultivars (ZS 758, black seeded; Zheda 622, yellow seeded). Vertical bars represent standard deviation from three independent replicates. Means followed by the same letters are not significantly different by Duncan's multiple range test (*P* < 0.05).

Glutathione reductase (GR) has ability to recycle the oxidized form of glutathione (GSSG) back to its reduced form (GSH) by maintaining higher GSH/GSSG ratio, which is required for the cellular protection against oxidative damage. Alterations in the activities of studied antioxidant enzymes were observed with the increase in Be levels (Figure 1). An increasing trend was found in SOD, POD, APX, and GR activities (except CAT) in both cultivars, that is, ZS 758 and Zheda 622. The better performance of ZS 758 as compared to Zheda 622 was reflected from their antioxidant activities under various levels of Be. The qRT-PCR analysis further confirmed the alterations in the above-mentioned antioxidant enzymes under various levels of Be (Figure 2). The transcript levels of studied antioxidant genes were significantly enhanced with the increase in Be levels (except CAT gene expression). The alterations in the transcript levels were insignificant at 100 and 200 *μ*M Be. The gene expression analysis in the leaves and roots of both* B. napus* cultivars was more prominent at 400 *μ*M Be level as compared to other treatments and control.

**Figure 3:**
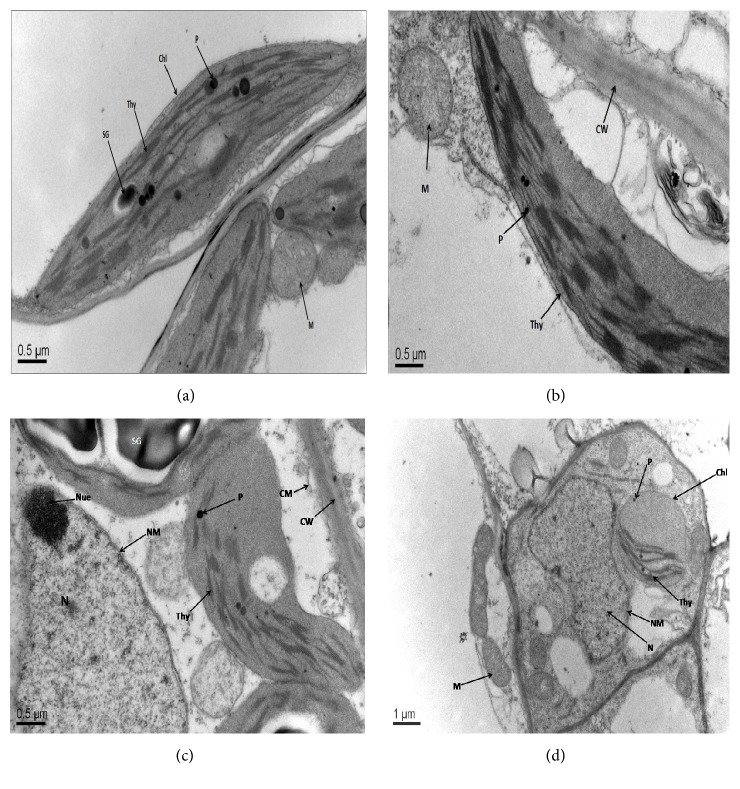
Electron micrographs of leaf mesophyll of 6-day-old seedlings of two cultivars of* Brassica napus* (ZS 758, black seeded; Zheda 622, yellow seeded) grown under control and 400 *μ*M Be. (a-b) leaf mesophyll cells of ZS 758 and Zheda 622 at control level, respectively, show well-developed cell wall (CW), chloroplasts (Chl), plastoglobuli (P), starch grain (SG), and mitochondria (M). (c) leaf mesophyll cell of ZS 758 at 400 *μ*M Be shows an unmatured nucleus (N) with nucleolus (Nue), disturbed nuclear membrane (NM), deshaped thylakoids (Thy), and small-sized plastoglobuli (P). (d) leaf mesophyll cell of Zheda 622 at 400 *μ*M Be shows damaged thylakoid membranes (Thy), disturbed nuclear membrane (NM), very small plastoglobuli (P), ruptured mitochondria (M), and chloroplast structures.

**Figure 4:**
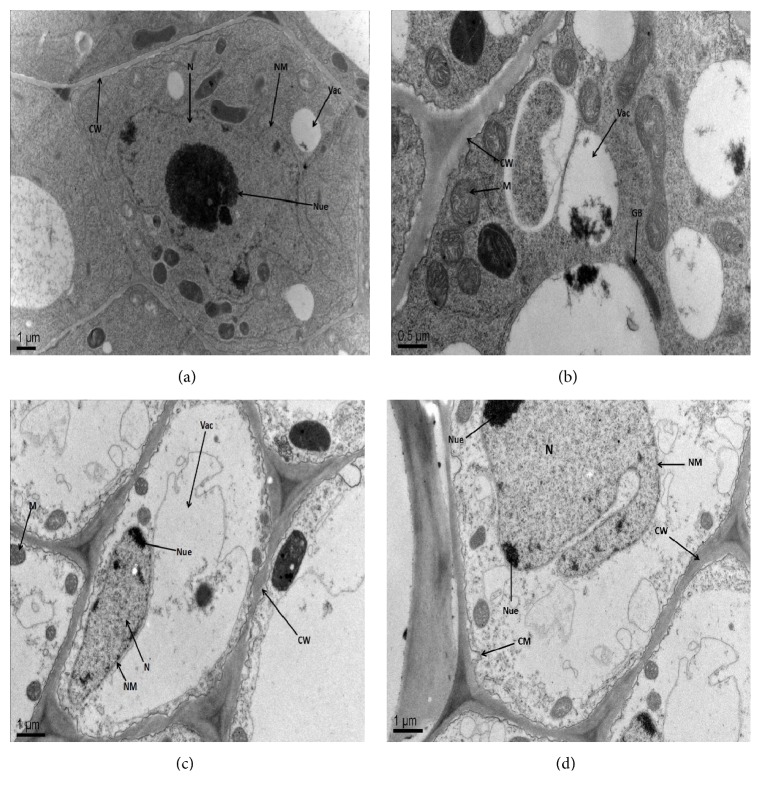
Electron micrographs of root cells of 6-day-old seedlings of two cultivars of* Brassica napus* (ZS 758, black seeded; Zheda 622, yellow seeded) grown at control and with 400 *μ*M Be. (a-b) root cells of ZS 758 and Zheda 622 at control level, respectively, show well-developed nucleus (N) with nucleolus (Nue), vacuole (Vac), nuclear membrane (NM), cell wall (CW), and well-developed mitochondria (M) as well as Golgi bodies. (c) root cell of ZS 758 at 400 *μ*M Be shows a damaged cell wall (CW), deshaped nucleus (N) with nucleolus (Nue), small-sized mitochondria (M), disturbed large vacuole (Vac), and disrupted nuclear membrane (NM). (d) root cell of Zheda 622 at 400 *μ*M Be shows a broken cell wall (CW), disturbed nucleus (N) with nucleolus (Nue), damaged cell membrane (CM), and nuclear membrane (NM).

The ultrastructural variations were observed in leaf mesophyll and root tip cells of two* B. napus* cultivars (cvs. ZS 758 and Zheda 622) under control and 400 *μ*M Be level (Figures 3 and 4). All the organelles in the leaf mesophyll and root tip cells were observed as well-developed and matured under control conditions (Figures 3(a) and 3(b)). However, higher Be level (400 *μ*M) showed markedly damaged leaf mesophyll (Figure 3) and root tip cells (Figure 4). At 400 *μ*M Be level, the alterations in leaf mesophyll cells were found including broken cell wall, damaged thylakoid membranes and chloroplast, deshaped and unmatured nucleus, and ruptured mitochondria. More damaging effects were revealed in Zheda 622 as compared to ZS 758 (Figures 3(c) and 3(d)). In roots, there were clear cell wall, rounded mitochondrial, nucleus with the well-developed nucleolus, and a clear nuclear membrane under control conditions (Figures 4(a) and 4(b)). Higher Be concentration (400 *μ*M) showed disrupt nuclear membrane, broken cell wall, damaged nucleus, and small size mitochondria (Figures 4(c) and 4(d)). More organelle damage was observed in Zheda 622 as compared to ZS 758. This showed that Zheda 622 was more sensitive to Be-toxicity than ZS 758.

## 4. Discussion

The accumulations of toxic metals in agriculture soils have become a major issue worldwide [43]. In plants, HMs cause oxidative stress that leads to cellular damage and ultimately inhibits the plant growth characteristics [44]. The present study was conducted to investigate the Be-induced physiochemical, oxidative injury and ultrastructural changes in two* B. napus *cultivars, that is, ZS 758 and Zheda 622. Results showed that shoot and root lengths were decreased gradually with the increase in Be concentrations in both* B. napus *cultivars (Table 1). These results are in accordance with the findings of Kopyra and Gwóźdź [45] and Atici et al. [46] that HM toxicity inhibits the plant growth parameters. Hopkins [47] and Encina and Becerra [25] further confirmed that higher concentrations of Be deteriorated the plant root length. Plant biomass (root, stem, leaf), fresh and dry weights, was considerably reduced in both cultivars (ZS758, Zheda 622) under Be stress, especially in Zheda 622 (Table 1). Plants are prone to damage with >1 ppm of ionized Be. In cabbage, the higher Be concentration caused 50% yield reduction corresponding to 3000 mg kg^−1^ in the roots and 6 mg kg^−1^ dry weight in the leaves [24]. Similarly, current study showed obvious reduction in root dry biomass at higher Be concentrations in both* Brassica* cultivars (Table 1).

A significant reduction in the chlorophyll contents was observed under higher Be concentrations (200 and 400 *μ*M), but lower Be concentration (100 *μ*M) showed insignificant reduction in the leaves of both* B. napus *cultivars (Table 2). These findings were in accordance with Küpper et al. [48] and Ali et al. [2] that Zn and Cd application declined the chlorophyll contents in* Arabidopsis* and oilseed rape, respectively. This might be due to the disturbances in the protein complexes and photosynthetic apparatus that decline the chlorophyll contents under metal stress [49]. Carotenoids serve as antioxidants by scavenging free radicals, reduce cell injury, and lessen the damage in chloroplast membrane induced by HMs [50]. Singh and Sinha [51] investigated a decrease in Rubisco activity that plays a key role in the reduction of pigment concentration, as observed in the current study (Table 2) which revealed that higher concentrations of Be reduced the total soluble protein (TSP) contents in both* B. napus *cultivars (Table 2). These findings are in line with the results of Gunes et al. [52] in which they found that HM stress declined TSP contents in* B. juncea.*

Malondialdehyde (MDA) is an important indicator of oxidative damage induced by metal stress [53]. MDA contents in leaves and roots were enhanced with the increase in Be concentrations in both* B. napus* cultivars (Table 3). Similar findings were observed by Romney et al. [26]; that is, higher Be stress increased the lipid peroxidation rate. The current study found higher production of ROS with the increase in Be concentrations in both* B. napus* cultivars (Table 3). The cultivar Zheda 622 was found more sensitive to ROS as compared to ZS 758, which is in accordance with the previous findings that tolerant plants showed less accumulation of ROS as compared to sensitive one [6]. Liu et al. [54] also reported an increase in H_2_O_2_ level in* Pteris vittata *L. and* Pteris ensiformis *L. This might be due to the cellular damage induced by HM stress that ultimately causes the cell death.

Plants have developed various strategies to scavenge the ROS and detoxify abiotic stress [55]. The induction of antioxidant defense system prevents the plants from oxidative damage [56]. In the present study, Be-toxicity triggered the antioxidant enzymatic defense system including SOD, POD, APX, and GR activities (except CAT) in both* B. napus* cultivars (Figure 1). An enhancement in SOD activity in the leaves and roots of both* B. napus* cultivars (ZS 758 and Zheda 622) was found with the increase in Be levels (Figures 1(a) and 1(b)), which confirms the results of previous study by Gupta et al. [57]. Similarly, the increase in POD activity was noted in both* B. napus* cultivars under Be stress (Figures 1(c) and 1(d)), which is similar to the investigations carried out by Qureshi et al. [58] in* Arabidopsis paniculata*. Furthermore, CAT activity was reduced with the increase of Be concentrations in both* B. napus *cultivars (Figures 1(e) and 1(f)). The reduction in CAT activity under Be stress might be due to enhanced H_2_O_2_ accumulation that results in its inactivation [59]. The activities of GR and APX were increased in both* B. napus* cultivars with the increase in Be concentrations (Figures 1(g)–1(j)). These findings were in line with Masood et al. [60] that HM stress improves the GR and APX activities in* H. annuus *and* B. juncea*. The increase in SOD, POD, APX, and GR activities proved that both cultivars have the ability to cope with HM stress and adapt themselves against Be stress by modulating the antioxidant defense system. This activated antioxidant defense system may assist plants in removing or scavenging the excess ROS production and hinder the lipid peroxidation [2]. Recently, Shah et al. [19] also suggested that Be-toxicity can alter enzyme activities by disturbing their metabolic functions. The seedlings of Zheda 622 have shown more oxidative stress due to the higher production of ROS under Be stress than those of black seeded cultivar ZS 758, which showed higher antioxidant activities (Figure 1).

The transcript levels of antioxidant genes including SOD, POD, GR, and APX were increased (decline in CAT gene) in the leaves and roots of both* B. napus* cultivars under Be stress (Figure 2). The upregulation of these above-mentioned genes was increased significantly in cultivar ZS 758 as compared to the sensitive cultivar Zheda 622. This upregulation in different genes suggested their direct involvement in defense-related mechanisms under Be stress that exhibited the Be-tolerance in* B. napus* cultivars. Besides, plants also triggered their nonenzymatic antioxidant system such as GSH and GSSG levels to strengthen their protection against stress conditions [61]. Results showed that both GSH and GSSG were increased with the increase in Be stress in the leaves and roots of both cultivars (Table S2). Similarly, Gill et al. [6] also investigated the protective role of GSH/GSSG ratio in reducing the damage induced by Cr stress in* B. napus*.

The ultrastructural alterations in leaf mesophyll and root tip cells were observed under different Be levels (Figures 3 and 4). Higher Be levels markedly damaged the thylakoids membrane, starch grain, plastoglobuli, mitochondrial, and chloroplast structures. More obvious alterations were observed in Zheda 622 as compared to ZS 758. Similar damage in the leaf mesophyll and root tip cell ultrastructures was investigated in* B. napus* against Cr stress [6]. In conclusion, the present study highlighted that both* B. napus* cultivars have shown different capability to face Be-toxicity. Our findings depicted that Be-toxicity had significantly declined the plant growth traits, biomass production, chlorophyll contents, and total soluble protein contents in both cultivars. The application of Be has caused oxidative damage by inducing ROS and MDA contents in a dose-dependent manner. The improvement in antioxidant enzyme activities including SOD, POD, APX, and GR (except CAT) was observed in both* B. napus* cultivars under Be stress. Antioxidant enzymes were further confirmed by gene expression analysis. The upregulation of the above-mentioned genes has suggested their direct involvement in defense mechanism under Be stress. Additionally, the increase in nonenzymatic antioxidants (GSH, GSSG) against studied Be levels showed plant protection against stress conditions. The electron microscopic study revealed that the ultrastructural damage in leaf mesophyll and root tip cells was more prominent in Zheda 622 as compared to ZS 758. These findings showed that Zheda 622 proved to be more sensitive cultivar than ZS 758. The present study would be of great interest to scientists working on phytoremediation and related areas. However, further investigations are required regarding Be-toxicity in soil-based environment.

## Figures and Tables

**Table 1:** Effects of different concentrations of beryllium (Be) on root length (cm), stem height, and biomass accumulation (g per 10 seedlings) in leaf, stem, and root tissues of 6-day-old seedlings of two *Brassica napus* cultivars.

Cultivar	Be conc. [*μ*M]	Shoot height	Root length	Leaf fresh weight	Leaf dry weight	Stem fresh weight	Stem dry weight	Root fresh weight	Root dry weight
ZS 758	0	4.64 ± 0.20^a^	4.40 ± 0.19^a^	1.45 ± 0.10^a^	0.067 ± 0.002^a^	0.48 ± 0.07^ab^	0.03 ± 0.001^a^	0.51 ± 0.08^a^	0.05 ± 0.002^a^
	100	4.42 ± 0.15^ab^	4.10 ± 0.17^b^	1.22 ± 0.09^b^	0.054 ± 0.002^b^	0.40 ± 0.06^bc^	0.02 ± 0.001^b^	0.42 ± 0.07^ab^	0.04 ± 0.002^b^
	200	3.95 ± 0.16^c^	3.50 ± 0.17^c^	0.86 ± 0.08^c^	0.035 ± 0.001^d^	0.32 ± 0.06^bc^	0.02 ± 0.0006^c^	0.28 ± 0.06^cd^	0.02 ± 0.001^c^
	400	3.21 ± 0.13^d^	2.53 ± 0.15^d^	0.58 ± 0.07^d^	0.017 ± 0.001^f^	0.21 ± 0.05^de^	0.01 ± 0.0001^d^	0.18 ± 0.05^de^	0.01 ± 0.001^d^

Zheda 622	0	4.62 ± 0.20^a^	4.44 ± 0.19^a^	1.43 ± 0.10^a^	0.065 ± 0.002^a^	0.51 ± 0.07^a^	0.03 ± 0.001^a^	0.47 ± 0.08^ab^	0.05 ± 0.005^a^
	100	4.32 ± 0.18^b^	3.90 ± 0.16^b^	1.16 ± 0.09^b^	0.050 ± 0.002^c^	0.38 ± 0.07^bc^	0.02 ± 0.0008^b^	0.38 ± 0.06^bc^	0.04 ± 0.002^b^
	200	3.86 ± 0.16^c^	3.27 ± 0.13^c^	0.78 ± 0.07^c^	0.030 ± 0.0015^e^	0.29 ± 0.06^cd^	0.02 ± 0.001^c^	0.24 ± 0.05^de^	0.02 ± 0.002^c^
	400	3.08 ± 0.14^d^	2.39 ± 0.13^d^	0.46 ± 0.06^d^	0.010 ± 0.001^g^	0.18 ± 0.04^e^	0.0087 ± 0.0001^d^	0.14 ± 0.04^e^	0.01 ± 0.001^d^

Means ± SD, *n* = 3. Values followed by different letters within a column are significantly different by Duncan's multiple range test (*P* < 0.05).

**Table 2:** Effects of different concentrations of beryllium (Be) on chlorophyll contents [mg g^−1^ (f.m.)] and leaf total soluble proteins (TSP) [mg g^−1^ (f.m.)] in cotyledons of 6-day-old seedlings of two *Brassica napus* cultivars.

Cultivar	Be conc. [*μ*M]	Chlorophyll a	Chlorophyll b	Carotenoid	TSP
ZS 758	0	35.78 ± 2.37^a^	65.84 ± 2.96^b^	47.70 ± 2.65^a^	2.44 ± 0.03^a^
	100	31.65 ± 2.27^b^	55.72 ± 2.50^c^	43.44 ± 2.77^b^	2.28 ± 0.02^c^
	200	23.64 ± 1.36^c^	33.43 ± 1.66^d^	31.49 ± 2.42^c^	2.02 ± 0.03^e^
	400	10.58 ± 1.07^e^	19.57 ± 0.83^f^	19.73 ± 1.90^d^	1.97 ± 0.01^f^

Zheda 622	0	37.5 ± 1.73^a^	69.82 ± 2.81^a^	51.74 ± 2.81^a^	2.39 ± 0.03^b^
	100	29.66 ± 1.84^b^	53.07 ± 2.81^c^	41.19 ± 2.65^b^	2.25 ± 0.02^c^
	200	18.30 ± 1.17^d^	28.46 ± 1.23^e^	28.76 ± 2.15^c^	2.07 ± 0.02^d^
	400	8.44 ± 1.13^e^	13.73 ± 0.83^g^	17.71 ± 1.49^d^	1.85 ± 0.02^g^

Means ± SD, *n* = 3. Values followed by different letters within a column are significantly different by Duncan's multiple range test (*P* < 0.05).

**Table 3:** Effects of different concentrations of beryllium (Be) on hydroxyl ion (^−^OH) [*μ*mol g^−1^ (f.m.)], hydrogen peroxide (H_2_O_2_) [*μ*mol g^−1^ (f.m.)], and malondialdehyde (MDA) [nmol mg^−1^ (protein)] contents in leaves and roots of 6-day-old seedlings of two *Brassica napus* cultivars.

Cultivar	Be conc. [*μ*M]	OH^−^ content		H_2_O_2_ content		MDA content	
		Leaf	Root	Leaf	Root	Leaf	Root
ZS 758	0	0.14 ± 0.002 ^g^	0.103 ± 0.001^c^	1.65 ± 0.02 ^g^	1.33 ± 0.02^g^	8.49 ± 1.24^d^	7.58 ± 1.20^d^
	100	0.15 ± 0.003^f^	0.105 ± 0.001^c^	1.78 ± 0.02^f^	1.43 ± 0.02^f^	10.61 ± 1.17^cd^	9.24 ± 1.28^d^
	200	0.18 ± 0.003^d^	0.113 ± 0.015^c^	1.99 ± 0.02^d^	1.62 ± 0.02^d^	14.59 ± 2.13^b^	13.43 ± 2.13^c^
	400	0.22 ± 0.004^b^	0.193 ± 0.015^b^	2.33 ± 0.03^b^	1.82 ± 0.03^b^	21.69 ± 2.95^a^	17.59 ± 1.73^b^

Zheda 622	0	0.14 ± 0.002 ^g^	0.099 ± 0.001^c^	1.68 ± 0.02^g^	1.29 ± 0.02^g^	7.80 ± 0.98^d^	7.62 ± 1.40^d^
	100	0.17 ± 0.004^e^	0.104 ± 0.002^c^	1.85 ± 0.02^e^	1.49 ± 0.02^e^	9.13 ± 1.80^d^	12.45 ± 1.00^c^
	200	0.21 ± 0.003^c^	0.114 ± 0.002^c^	2.09 ± 0.02^c^	1.69 ± 0.04^c^	13.43 ± 1.92^bc^	16.34 ± 1.89^b^
	400	0.25 ± 0.004^a^	0.260 ± 0.02^ca^	2.56 ± 0.03^a^	1.98 ± 0.03^a^	19.77 ± 1.90^a^	24.51 ± 1.85^a^

Means ± SD, *n* = 3. Values followed by different letters within a column are significantly different by Duncan's multiple range test (*P* < 0.05).

## Supplementary Materials

Supplementary Materials Table S1: oligonucleotide sequences of antioxidants related transcripts used in qRT-PCR analysis. Table S2: effects of different concentrations of beryllium (Be) on GSH and GSSG [*µ*molg^−1^ (d.m.)] contents in the leaves and roots of two* Brassica napus* cultivars.
