# Supplementary material for: Beryllium Stress-Induced Modifications in Antioxidant Machinery and Plant Ultrastructure in the Seedlings of Black and Yellow Seeded Oilseed Rape
Source: Biomed Res Int. 2018 Mar 21;2018:1615968. doi: 10.1155/2018/1615968 (PMC5884399; doi:10.1155/2018/1615968)
Supplement: Supplementary Materials — Table S1: oligonucleotide sequences of antioxidants related transcripts used in qRT-PCR analysis. Table S2: effects of different concentrations of beryllium (Be) on GSH and GSSG [µmolg−1 (d.m.)] contents in the leaves and roots of two Brassica napus cultivars. [file 1615968.f1.docx]

**Table S1** Oligonucleotide sequences of antioxidants related transcripts used in qRT-PCR analysis.

| Sr. No. | Gene description | Forward | Reverse |
| --- | --- | --- | --- |
| 1 | Super oxide dismutase(SOD) | ACGGTGTGACCACTGTGACT | GCACCGTGTTGTTTACCATC |
| 2 | Peroxidase (POD) | ATGTTTCGTGCGTCTCTGTC | TACGAGGGTCCGATCTTAGC |
| 3 | Catalase (CAT) | TCGCCATGCTGAGAAGTATC | TCTCCAGGCTCCTTGAAGTT |
| 4 | Ascorbate peroxidases(APX) | ATGAGGTTTGACGGTGAGC | CAGCATGGGAGATGGTAGG |
| 5 | Glutathione reductase (GR) | AAGCTGGAGCTGTGAAGGTT | AGACAGTGTTCGCAAAGCAG |
| 6 | Actin | TTGGGATGGACCAGAAGG | TCAGGAGCAATACGGAGC |

**Table S2**Effects of different concentrations of[beryllium](https://en.wikipedia.org/wiki/Beryllium)(Be) on GSH and GSSG [µmolg^-1^ (d.m.)] contents in the leaves and roots of two *Brassica napus* cultivars.

| Cultivar | Be Conc.  [μM] | GSH contents | | GSSG contents | |
| --- | --- | --- | --- | --- | --- |
|  |  | Leaf | Root | Leaf | Root |
| ZS 758 | 0 | 98.48 ± 3.06cd | 42.43 ± 2.38f | 0.19 ± 0.01cd | 0.022 ± 0.01d |
|  | 100 | 104.19 ± 3.71c | 53.71 ± 2.33e | 0.22 ± 0.01c | 0.07 ± 0.02c |
|  | 200 | 115.45 ± 4.12b | 70.67 ± 2.78c | 0.29 ± 0.02b | 0.15 ± 0.02b |
|  | 400 | 127.63 ± 5.17a | 98.91 ± 3.38a | 0.4 ± 0.02a | 0.22 ± 0.02a |
| Zheda 622 | 0 | 93.72 ± 3.58d | 45.52 ± 2.33f | 0.18 ± 0.01d | 0.01 ± 0.0d |
|  | 100 | 99.48 ± 3.74cd | 52.91 ± 2.57e | 0.22 ± 0.02c | 0.06 ± 0.01c |
|  | 200 | 111.61 ± 4.22b | 63.49 ± 3.00d | 0.31 ± 0.02b | 0.13 ± 0.02b |
|  | 400 | 117.82 ± 5.12b | 82.19 ± 3.36b | 0.42 ± 0.03a | 0.2 ± 0.02a |

Means ± SD, n = 3. Values followed by different letters within a column are significantly different by Duncan's multiple range test (*P<0.05*).
